# A Multicomponent Educational and Rehabilitation Approach in Optimizing Outcomes During the Poststroke Subacute Phase

**DOI:** 10.15388/Amed.2024.31.2.20

**Published:** 2024-12-04

**Authors:** Alma Glinac, Osman Sinanović

**Affiliations:** 1 University Clinical Center Tuzla, Bosnia and Herzegovina; 2 Medical Faculty, University of Tuzla, Bosnia and Herzegovina; Sarajevo School of Medicine, University of Sarajevo School of Science and Technology, Bosnia and Herzegovina

**Keywords:** cognitive recovery, functional independence, motor function recovery, multicomponent therapy, stroke rehabilitation, Raktažodžiai: kognityvinis atkūrimas, funkcinis nepriklausomumas, motorinių funkcijų atkūrimas, daugia-komponentė terapija, insulto reabilitacija

## Abstract

**Background:**

Poststroke patients often experience cognitive impairments, motor weakness, and difficulties in daily activities. A multicomponent educational-rehabilitation approach offers a holistic treatment by integrating cognitive and motor training with relaxation, adaptive skills training, and motivational components. Despite its potential benefits, there is limited evidence supporting its effectiveness during the subacute stroke phase. This study evaluates the impact of this approach on cognitive functions, upper extremity motor skills, and daily activity independence during the subacute phase.

**Materials and Methods:**

In a prospective, randomized clinical trial, 40 patients participated in a 20-session multicomponent educational-rehabilitation program consisting of 45-minute sessions, five days a week. This program combined cognitive and motor training with additional components such as relaxation and patient education. The control group (N=30) received standard physical therapy, including electrotherapy and speech therapy as needed. Assessments were conducted at admission, after the final session for the experimental group, and one month later for the control group.

**Results:**

The experimental group demonstrated significantly greater improvements in cognitive abilities, upper extremity functionality, and daily activity independence compared to the control group (p < 0.001).

**Conclusion:**

The multicomponent educational-rehabilitation approach significantly enhances cognitive function, motor skills, and daily activity independence during the subacute phase of stroke recovery. These findings suggest that this approach offers superior recovery outcomes compared to standard care, warranting further investigation into its long-term effects.

## Abstract (it)

**Įvadas:**

Po insulto pacientai dažnai patiria kognityvinius sutrikimus, motorinį silpnumą ir sunkumus atlikdami kasdienes veiklas. Daugiakomponentis edukacinės reabilitacijos metodas siūlo holistinį gydymą, integruojant kognityvinį ir motorinį treniravimą su atsipalaidavimo, adaptacinių įgūdžių treniravimo ir motyvacijos komponentais. Nepaisant metodo galimos naudos, yra nedaug įrodymų, patvirtinančių jo efektyvumą subakutinėje insulto fazėje. Šis tyrimas vertina šio metodo poveikį kognityvinėms funkcijoms, viršutinių galūnių motoriniams įgūdžiams ir nepriklausomumui kasdienėje veikloje subakutinėje fazėje.

**Medžiaga ir metodai:**

Perspektyviajame, atsitiktinės atrankos klinikiniame tyrime dalyvavo 40 pacientų. Prieš tai jie dalyvavo 20 sesijų daugiakomponentėje edukacinės reabilitacijos programoje, kuri truko 45 minutes, penkis kartus per savaitę. Ši programa sujungė kognityvinį ir motorinį treniravimą su papildomais komponentais, tokiais kaip atsipalaidavimas ir pacientų edukacija. Kontrolinė grupė (N = 30) gavo standartinę fizinę terapiją, įskaitant elektroterapiją ir kalbos terapiją, jei to reikėjo. Vertinimai buvo atliekami pradedant tyrimą, po paskutinės sesijos eksperimentinei grupei ir po mėnesio kontrolinėje grupėje.

**Rezultatai:**

Eksperimentinės grupės kognityvinių gebėjimų, viršutinių galūnių funkcionalumo ir nepriklausomumo kasdienėje veikloje pagerėjimas buvo didesnis, palyginti su kontroline grupe (p < 0,001).

**Išvada:**

Daugiakomponentis edukacinės reabilitacijos metodas žymiai pagerina kognityvines funkcijas, motorinius įgūdžius ir nepriklausomumą kasdienėje veikloje subakutinėje insulto atkūrimo fazėje. Šie duomenys rodo, kad šis metodas siūlo geresnius atkūrimo rezultatus, palyginti su standartine priežiūra, ir verta toliau tyrinėti jo ilgalaikį poveikį.

## Introduction

Stroke, also known as cerebrovascular accident (CVA), is a pathological condition caused by dysfunction of cerebral blood flow, resulting in brain damage. It is defined as a sudden brain attack causing partial or complete brain dysfunction due to blocked or ruptured blood vessels in the brain [1]. The primary symptom of stroke is hemiparesis. Additionally, stroke adversely affects sensations, motor functions, perception, cognition, and language, depending on the location, etiology, and volume of the infarct [2]. Up to 20 to 80% of stroke survivors experience difficulties in cognitive functioning [3,4], while 50 to 70% exhibit motor deficits in the affected upper extremity [5], as well as perceptual and/or sensory impairments. Mood is often compromised, with 5 to 63% of patients developing mood disorders [6], significantly affecting their daily functionality and quality of life. Statistically, stroke stands out as the second most common cause of death and disability among adults globally [7], with approximately 15 million cases annually worldwide, and about 30% of patients develop permanent disabilities [8].

Rehabilitation after stroke can be considered a process of (motor) relearning [9,10], aimed at restoring skilled movement through training that promotes neuroplastic changes [9,11-14]. However, even with the application of modern standards of care, motor recovery often remains incomplete [15]. In long-term, 30% of cognitive [16-19] and 15–30% of motor deficits persist [20], highlighting the urgent need for new therapeutic strategies [21].

After tissue necrosis, there is currently no medical intervention that can fully restore lost tissue. Therefore, restorative therapeutic interventions have become the standard of care [22,23]. A multi-component educational-rehabilitative approach is based on the concepts of brain neuroplasticity [24] and motor learning [25], providing a holistic approach [26] to the recovery process. This approach integrates various therapeutic techniques and interventions aimed at improving patients’ functional abilities and quality of life. It consists of multiple components, including cognitive training, motor training of the affected upper extremity, relaxation, training for independence and self-help, education and counseling, motivational training, and metacognition. The core of the treatment incorporates the integration of cognitive and motor training, while other components are applied through contextualized interactions, tailored to the individual needs and condition of each patient. The integration of different therapeutic modalities enables synergistic effects that can improve the functionality and quality of life of patients 27].

This study aimed to evaluate the effects of a multicomponent educational-rehabilitative therapeutic approach to the restitution of cognitive functions, motor skills of the affected upper extremity, and functional independence in daily activities in the subacute phase after stroke. The results of this research are expected to provide a deeper understanding of the efficacy of the proposed therapeutic approach and offer guidelines for further improvement of rehabilitation strategies for patients in the subacute phase of stroke.

## Materials and Methods

### Study Design

This prospective, randomized clinical trial was conducted to evaluate the effectiveness of a multicomponent educational-rehabilitative treatment in stroke patients. Seventy patients who had experienced a stroke and were undergoing rehabilitation at the Clinic for Physical Medicine and Rehabilitation, University Clinical Center Tuzla, were included in the study. The study protocol received approval from the Ethics Committee of the University Clinical Center Tuzla (approval number: 02-09/2-48/18).

### Inclusion Criteria

Participants were included if they met the following criteria: (1) aged 40–60 years, (2) confirmed stroke diagnosis via CT or MRI, (3) first-ever stroke, (4) stroke occurrence within six months prior to enrollment, (5) absence of preexisting physical disabilities from other neurological, orthopedic, or rheumatological conditions, (6) no psychiatric disorders, (7) no disturbances of consciousness, (8) preserved ability to understand and follow simple commands, (9) absence of aphasia, and (10) provided voluntary informed consent.

### Study Participation

The 70 eligible stroke patients were randomly assigned to either the experimental group (N=40) or the control group (N=30) using simple randomization. A unique identifier was allocated to each patient for study tracking. Randomization was conducted using a card-drawing method, where patients selected a card marked “E” for the experimental group or “C” for the control group.

Baseline assessments were conducted to evaluate cognitive and motor abilities, as well as functional independence in activities of daily living. Sociodemographic data (age, gender, education level, marital status) and disease-specific factors (stroke type, brain lesion location) were recorded.

### Interventions

Both groups received standard physical therapy, including electrotherapy and individualized kinesiotherapy, with additional speech therapy as needed. The experimental group also participated in a multicomponent educational-rehabilitative program consisting of 20 sessions, each lasting 45 minutes, administered five days per week. Treatment priorities were identified based on initial assessments, with goals and specific therapeutic methods defined accordingly.

### Outcome Measures

Assessments were conducted at baseline, immediately after the last session for the experimental group, and one month later for the control group.

- Cognitive function was assessed using the Montreal Cognitive Assessment (MoCA), which evaluates eight domains: visuospatial/executive functions, naming, memory, attention, language, abstraction, delayed recall, and orientation. The maximum score is 30, with a score of 26 or higher considered normal. Participants with less than 12 years of education received an additional point to their total score. Testing was conducted at two intervals: upon admission and after one month [28.
- Motor function of the affected hand was assessed using the Motor Evaluation Scale for Upper Extremity in Stroke Patients (MESUPES), which comprises 17 items divided into hand function (eight items, scores 0–5) and grasp function (nine items, scores 0–2). The maximum score on the MESUPES scale is 58 points [29].
- Functional independence was evaluated using the Functional Independence Measure + Functional Assessment Measure (FIM+FAM), a 30-item scale divided into motor (18 items) and cognitive (12 items) subscales. Scores range from 1 (complete dependence) to 7 (complete independence) in performing activities of daily living [30].

### Multicomponent Educational-Rehabilitative Approach

Upon a physiatrist’s recommendation for an educational-rehabilitative treatment, the collection of anamnesis data on the current state of the patients began. Information regarding the stroke, clinical presentation, symptoms, causes, duration of the rehabilitation treatment, and factors contributing to improvement were provided to patients clearly and understandably, emphasizing optimism, hope, and a vision of recovery. A thorough assessment using relevant evaluation tests was conducted, and the priority treatment area was determined based on identified deficits. Treatment goals and methods were set accordingly, and patients were continuously given opportunities to express their feelings related to the illness, reactions to the diagnosis, and therapy. It was explained that they are active participants in the stroke recovery process, and their active role is crucial for achieving recovery.

The multicomponent educational-rehabilitative approach represents a synergy of various therapeutic techniques and interventions within a holistic framework aimed at ensuring comprehensive recovery after a stroke. This approach is based on fundamental principles of brain neuroplasticity and motor learning and advocates for a complex rehabilitation approach that promotes adaptation and regeneration of brain structures while encouraging optimal functioning of motor abilities. The holistic approach allows for personalized intervention tailored to the specific needs of each patient, focusing on all relevant aspects contributing to their difficulties. This includes cognitive training, development of motor skills of the affected upper limb, relaxation techniques to reduce stress and tension, independence and self-care training, education and counseling, motivational training, and metacognition. The core elements of the treatment integrate cognitive and motor training, while other components are applied through contextualized interactions, adapting to the individual needs and conditions of each patient.

### Cognitive Training

Cognitive training was conducted in several phases. The initial phase included tasks such as gesture imitation, gesture comprehension, execution of simple and complex tasks, and body part identification. The second phase focused on enhancing visuospatial abilities to improve visual perception and skills, following a detailed evaluation of fundamental visual functions, including visual acuity, visual field, eye movement, tracking ability, and visual attention. Techniques employed during this phase included the development of visuospatial skills, visual discrimination, visual memory, visual closure, visual figure/ground perception, and visuomotor integration. Therapeutic activities were designed to gradually increase task difficulty to enhance patients’ visuoperceptual skills. The third phase targeted improvement in attention, concentration, orientation, general memory, visual and auditory memory, sequential thinking, and reasoning. In the fourth phase, reading and writing exercises were conducted to further contribute to cognitive development [27].

### Training Motor Skills of the Affected Arm

The rehabilitation of a hemiplegic arm following a stroke focused on promoting neuroplasticity and functional recovery through a gradual extension of the range of motion, muscle strengthening exercises, and essential activities such as manipulating objects of various sizes. The therapeutic approach included proprioceptive exercises to improve limb position awareness in space and coordination exercises to foster synergy between the affected arm and other body parts, tailored to each patient’s specific needs. The motor reeducation method according to Bojanin (1985) emphasized the development of basic and advanced motor skills and the functional use of the affected arm through activities such as object manipulation, throwing, and catching, with a particular focus on enhancing fine motor coordination of the fingers and thumb [27,31].

### Relaxation

Patients were educated on relaxation techniques to manage mood changes, alleviate mood disorders, regulate negative emotions, increase energy and productivity, and improve concentration and attention. The relaxation exercises included abdominal breathing and breath-calming exercises [32].

### Independence and Self-Care Training

Strategies to overcome limitations in stroke survivors involved learning new ways to perform daily activities, including the use of special techniques (e.g., one-handed methods), adaptive devices (e.g., utensils with thicker handles), and home modifications (e.g., recommendations for installing ramps and handrails). The goal was for patients to achieve the highest possible level of independence and improve their quality of life despite existing functional losses.

### Patient Education and Counseling

Education and counseling provided patients with relevant information about their illness, encouraged understanding of their health condition, and promoted healthy lifestyle choices. The aim was to empower patients, improve health outcomes, and reduce unpleasant experiences during hospital stays, including lowering levels of pain and anxiety [33-35].

### Motivational Training

The goal of motivational training was to encourage active patient participation in rehabilitation by promoting positive changes in health behavior, engaging in deep conversations about their lives and fulfilling activities, and strengthening confidence in the recovery process. Additionally, the purpose of rehabilitation was emphasized, relevant goal-setting for each patient was conducted, detailed information about the rehabilitation process was provided, and cultural and individual differences of the patients were considered [36].

### Metacognition

Patients were encouraged to use metacognitive strategies such as self-questioning, paraphrasing, explaining, and goal-setting. These strategies aimed to increase awareness of difficulties, set improvement goals, and enhance performance in daily activities, thereby reducing disability [37].

## Statistical Analyses

Statistical analysis was performed using SPSS (Statistical Package for the Social Sciences) for Windows, version 23. Results are presented as means and standard deviations. Chi-square test and Fisher’s exact test were used to assess differences among categorical variables. Independent samples t-test was applied to compare differences between independent groups assuming normal distribution. Differences were considered significant at a level of less than 0.05.

## Results

The study included a total of 70 participants who met the inclusion criteria, with 40 in the experimental group (mean age 53.3 ± 5.47 years) and 30 in the control group (mean age 53.9 ± 2.86 years); no participants withdrew from the study after signing informed consent. Clinical and sociodemographic characteristics of the patients are presented in Table 1. No significant differences between groups were observed for these parameters.

**Table 1:** Clinical Profile and Demographic Characteristics of Stroke Patients in Experimental and Control Groups

Characteristics	Experimental group	Control group	P
	N=40	N=30	
Gender (Male/Female)	24/16	16/14	0.577^a^
Education (Elementary/High School/Higher Education)	13/27/0	15/14/1	0.139^a^
Employment status (employed/unemployed/retired)	13/18/9	9/16/5	0.752^a^
Marital status (Average/Below average)	38/2	27/3	0.645^b^
Marital status (married/other)	32/8	23/7	0.737^a^
Type of stroke (ischemic/hemorrhagic)	34/6	28/2	0.752^b^
Site of lesion (right/left hemisphere)	21/19	22/8	0.076^b^

^a^ Chi square test; ^b^ Fisher´s exact test

The average time from stroke onset to the initial educational-rehabilitation assessment in the experimental group was 39 days (the earliest assessment on day 6, the latest on day 200), while in the control group, the average time was 45 days (most frequent assessment on day 23, minimum 10 days, maximum 167 days). Statistical analysis revealed no significant differences between the groups in the time from stroke onset to inclusion in the initial educational-rehabilitation assessment (t = -0.581, df = 68, p = 0.563).

### Cognitive Impairment and Recovery

A high prevalence of cognitive impairment was observed, with 97.5% of patients in the experimental group and 96.67% in the control group showing impairment (Table 2).

**Table 2:** Review of the Existence of Cognitive Impairment in Stroke Patients

MoCA		Experimental group		Control group	
		Initial assessment	Final assessment	Initial assessment	Final assessment
	Score	N (%)	N (%)	N (%)	N (%)
Without cognitive impairment	26–30	1 (2.5)	25 (62.5)	1 (3.3)	2 (6.6)
Mild cognitive impairment	18–25	20 (50.0)	12 (30.0)	15 (50.0)	17 (56.7)
Moderate cognitive impairment	10–17	15 (37.5)	3 (7.5)	10 (33.3)	10 (33.3)
Severe cognitive impairment	9–0	4 (10.0)	0 (0.0)	4 (13.4)	1 (3.4)

MoCA – Montreal Cognitive Assessment test.

Average values of MoCA test results did not show significant differences between the experimental and control groups during the initial assessment. However, at final measurements, the average values of results were significantly higher in the experimental group (Table 3).

**Table 3:** Display of the difference in mean scores on the MoCA test between groups at the initial and final measurements

MoCA	Initial assessment		P	Final assessment		P
	Experimental group	Control group		Experimental group	Control group	
	x (SD)	x (SD)		x (SD)	x (SD)	
TOT	16.32 (5.34)	17.70 (5.61)	0.305	25.27 (4.54)	18.93 (4.98)	<0.001
VS	2,30 (1.66)	1.93 (1.72)	0.372	3.50 (1.45)	1.90 (1.68)	<0.001
NAM	2.40 (0.87)	2.73 (0.52)	0.067	3.00 (0.00)	2.90 (0.30)	0.042
ATT	3.57 (1.93)	3.83 (1.74)	0.566	4.97 (1.12)	4.20 (1.62)	0.021
LAN	0.97 (0.86)	1.26 (0.86)	0.167	2.45 (0.84)	1.33 (0.88)	<0.001
ABS	0.40 (0.49)	0.60 (0.62)	0.139	1.50 (0.67)	0.76 (0.67)	<0.001
DEL	1.35 (1.02)	1.76 (1.10)	0.108	3.57 (1.12)	1.83 (1.40)	<0.001
ORI	5.10 (0.98)	5.10 (1.21)	1.00	5.90 (0.26)	5.50 (0.68)	0.001

MoCA – Montreal Cognitive Assessment test; TOT – total score; VS – visuospatial and executive functions; NAM – naming; ATT – attention; LAN – language; ABS – abstract thinking; DEL – delayed recall; ORI – orientation.

The results of the statistical analysis at the initial measurement indicated no statistically significant differences in cognitive functioning between the experimental and control groups. This finding confirms the homogeneity of the sample and ensures objectivity in evaluating the effects of the intervention.

Following the intervention, the results revealed statistically significant differences in favor of the experimental group (p < 0.001). An analysis of the results presented in Table 3 showed that the average MoCA test scores at the final measurement were significantly higher in the experimental group compared to the control group. The differences in score changes between the groups were statistically significant for overall cognitive functioning (TOT: t=5.543, p<0.001), visuospatial and executive abilities (VS: t=4.257, p<0.001), naming (NAM: t=2.078, p=0.042), attention (ATT: t=2.359, p=0.021), language (LAN: t=5.362, p<0.001), abstract thinking (ABS: t=4.471, p<0.001), delayed recall (DEL: t=6.341, p<0.001), and orientation (ORI: t=3.597, p=0.001).

These findings highlight the significant impact of the intervention on cognitive functioning in the experimental group, further confirming the effectiveness of the applied educational-rehabilitation approach.

### Motor Function of the Affected Upper Extremity and Recovery

The motor function of the affected upper extremity in the experimental and control groups at the initial and final time points is presented in Table 4.

**Table 4:** Comparing Group Differences in Average MESUPES Item Values

MESUPES	Initial assessment		P	Final assessment		P	
	Experimental group	Control group		Experimental group	Control group		
	x (SD)	x (SD)		x (SD)	x (SD)		
TOTM	22.50 (11.59)	20.90 (10.02)	0.535	38.80 (19.17)	25.83 (12.11)		0.002
HF	20.00 (8.99)	19.43 (8.08)	0.786	28.07 (13.02)	22.86 (8.97)		0.064
FF	1.95 (2.59)	1.2 (2.28)	0.212	6.62 (4.90)	2.1 (2.84)	<0.001	
FT	0.65 (1.14)	0.26 (0.63)	0.104	3.27 (2.56)	0.83 (1.26)	<0.001	

TOTM – Total MESUPES; HF – hand function; FF – finger function; FT – functional tasks.

At the initial assessment, there were no statistically significant differences in the motor function of the affected upper extremity between the experimental and control groups of participants. This finding suggests that these two groups were initially comparable in terms of motor functioning of the affected upper extremities, ensuring the homogeneity of the sample at the start of the study.

Regarding the analysis of change in motor function of the affected upper extremity at the final assessment, results indicate significant improvements in the experimental group compared to the control group in overall motor function of the affected upper extremity (TOTM: t=3.247; p=0.002), finger function (FF: t=4.509; p<0.001), and performance of functional tasks (FT: t=4.796, p<0.001). For motor function of the affected hand (HF: t=1.879; p=0.064), better performance was observed posttreatment, although the differences were not statistically significant.

These findings highlight the significant positive effects of the experimental treatment on the motor functionality of the affected upper extremity in stroke patients, with the results demonstrating statistically significant differences after the intervention (p < 0.001). This reinforces the efficacy of the treatment while ensuring the objectivity of the analyses, with no significant differences at baseline between the experimental and control groups.

### Functional independence in daily activities

The depiction of functional independence in daily activities, measured by the FIM+FAM test, in the experimental and control groups at two time points, initial and final, is presented in Table 5.

**Table 5:** Comparing Group Differences in Average FIM+FAM Item Values

FIM+FAM	Initial assessment		P	Final assessment		P
	Experimental group	Control group		Experimental group	Control group	
	x (SD)	x (SD)		x (SD)	x (SD)	
TOTFF	98.55 (23.14)	96.80 (24.34)	0.760	145.40 (22.43)	112.10 (24.10)	<0.001
TOTFIM	68,05 (25.44)	61.90 (17.26)	0.258	96.77 (16.73)	75.83 (17.40)	<0.001
TOTFAM	35.37 (6.8)	34.90 (7.79)	0.787	48.62 (6.24)	36.26 (7.37)	<0.001
SCAR	27.10 (5.56)	27.90 (5.71)	0.558	39.20 (5.73)	32.90 (5.78)	<0.001
SCON	8.97 (2.34)	8.96 (2.96)	0.990	11.50 (2.02)	10.13 (2.23)	0.009
TRAN	9.45 (3.24)	8.86 (2.40)	0.411	13.80 (3.49)	11.66 (2.78)	0.008
LOKO	4.45 (2.62)	4.40 (2.07)	0.932	8.92 (2.88)	7.53 (2.50)	0.038
COMM	24.02 (4.88)	22.50 (5.35)	0.219	31.55 (3.58)	23.80 (4.97)	<0.001
PSYA	10.22 (3.64)	9.70 (4.25)	0.580	17.32 (3.30)	10.50 (4.04)	<0.001
COGF	14.07 (4.71)	14.46 (5.13)	0.742	23.10 (4.09)	15.56 (4.46)	<0.001

TOTFF - Total FIM+FAM; TOTFIM - Total FIM; TOTFAM – Total FAM; SCAR – Self-care; SCON – Sphincter control; TRAN – Transfers; LOKO – Locomotion; COMM – Communication; PSYA – Psychosocial adaptation; COGF – Cognitive functioning.

At the initial assessment, no statistically significant differences were observed in the level of functional independence in daily activities between the experimental and control groups, indicating initial homogeneity between the groups. This ensures the objectivity of the analyses and confirms that the sample was comparable at the outset of the study.

However, following the intervention, the results clearly demonstrate statistically significant improvements in the experimental group compared to the control group across all assessed aspects of functional independence. Statistically significant differences were observed in total FIM+FAM (TOTFF: t=5.953; p<0.001), total FIM (TOTFIM: t=5.093; p<0.001), total FAM (TOTFAM: t=7.579; p<0.001), self-care (SCAR: t=4.530; p<0.001), sphincter control (SCON: t=2.670; p=0.009), transfers (TRAN: t=2.751; p=0.008), locomotion (LOKO: t=2.112; p=0.038), communication (COMM: t=7.580; p<0.001), psychosocial adjustment (PSYA: t=7.766; p<0.001), and cognitive functioning (COGF: t=7.331; p<0.001). These findings unequivocally suggest that the experimental treatment significantly enhanced the functional independence of patients in performing daily activities.

## Discussion

The multicomponent educational-rehabilitation approach implemented in the subacute phase of stroke integrates various therapeutic techniques and interventions through a holistic approach addressing different aspects of recovery. This comprehensive approach includes cognitive training to improve cognitive functioning, motor skills training to restore upper limb motor abilities, relaxation techniques to reduce stress and tension, adaptive skills training to achieve independence and socialization, education and counseling for disease understanding and promotion of healthy habits, motivational training to encourage participation in rehabilitation, and metacognition to increase awareness of difficulties and goal-setting. The foundation of the treatment incorporates the integration of cognitive and motor training, while other components are applied through contextualized interactions tailored to individual patient needs and conditions.

In this study, stroke survivors faced the greatest challenges in abstract thinking, delayed recall, language, visuospatial abilities, and executive functions, while they encountered fewer difficulties in orientation. Similar findings have been reported in numerous studies confirming the high prevalence of cognitive impairments following stroke, including memory deficits, apraxia, attention deficits, impaired executive function, difficulties in calculation, speech, and constructional abilities [38-42]. The higher prevalence of cognitive impairment in this study compared to previous research may be explained by the presence of risk factors such as low premorbid cognitive abilities, stroke severity, and comorbidities [43]. Similar studies have shown that individuals with higher education have greater cognitive reserve and better cognitive recovery [44].

In our study, patients who received the multicomponent educational-rehabilitation treatment demonstrated significant improvement across all cognitive domains. Conversely, the control group did not show significant improvements; instead, there was a descriptive decline in visuospatial abilities and executive functions. These results are consistent with existing literature suggesting that treatments aimed at improving cognitive functions should be specifically designed with a focus on mental processes. Standard motor therapy typically focuses on enhancing physical abilities and coordination, but does not necessarily include exercises and activities directly related to cognitive processes [45]. Such an approach reduces the potential for cognitive improvement and disrupts the harmonious functional recovery that arises from the synergy between motor and cognitive activation. Our findings confirm previous research indicating that multidimensional cognitive therapy is more effective than therapies targeting a single domain [45,46]. Furthermore, the effects of cognitive treatment are more pronounced when combined with motor training [47].

Five studies examined the impact of cognitive impairment on functional outcomes and activities of daily living [48-52]. Lower performance in cognitive functions is positively associated with greater dependency on assistance in instrumental activities of daily living [53]. Information processing deficits represent a significant issue poststroke as they are linked to motor control during the execution of daily activities [44,54,55]. Executive function impairment poststroke is associated with reduced quality of life [56], disability in activities of daily living [57] and increased mortality [58].

Motor impairments of the upper extremities are a common consequence of stroke, affecting approximately 50–70% of patients [5]. According to Mirshoja et al. (2015), 65% of stroke survivors do not exhibit the ability to use their hemiparetic hand in routine activities [59]. Additionally, Lum et al. (2009) reported that about 38% of participants perceived the loss of hand function as the most challenging motor impairment they faced [60]. Common impairments include reduced motor ability and limitations in functional use of the paretic hand, caused by muscle weakness or paralysis, abnormal muscle tone, musculoskeletal issues, and coordination disorders [61]. This study demonstrates that stroke significantly impacts the motor functioning of the affected upper extremity, hand functionality, finger mobility, and performance of functional tasks in both examined groups.

The results of this study are consistent with previous research documenting impaired proximal control of the hand in individuals with hemiparesis, as well as restricted range of motion, coordination, and the discoordination between reaching and grasping movements [60]. Additionally, weakness in finger extensor grasp has been noted [62], resulting in loss of motor control.

Implemented multicomponent educational-rehabilitative treatment resulted in significantly improved motor performance of the affected upper extremity in the experimental group compared to the control group, particularly in overall motor function of the affected arm, finger function, and performance of functional tasks. Although better hand function was observed posttreatment, these differences were not statistically significant. Considering that patients in the control group did not show significant improvements in cognition, these findings align with literature evidence suggesting the impact of cognitive dysfunction on motor learning and rehabilitation outcomes [63,64]. Eschweiler et al. (2021) demonstrated that combined motor and cognitive treatment accelerates the recovery of motor and cognitive functions more effectively than motor training alone [21]. According to Chodosh et al. (2010), higher-order cognitive functions such as organization, problem-solving, and memory are linked to physical dependency in stroke survivors [65]. During rehabilitation, patients with impaired cognitive abilities may not effectively engage cognitive functions necessary for motor learning [66].

Limitations in hand and finger function pose significant challenges poststroke, impairing daily activities of patients [67]. Activities requiring bilateral use of upper extremities become impossible for stroke survivors due to the inability to use the paretic arm [68]. Even mild impairments in upper extremity function lead to significant limitations in daily functioning [60].

Although the results of this study are promising, it is important to emphasize that effective strategies for prevention and treatment, including a healthy lifestyle, modern therapies, and secondary prevention, contribute to better recovery and reduced mortality following stroke [69,70]. This multicomponent educational-rehabilitative approach demonstrates significant efficacy in improving cognitive and motor functions, particularly in patients who have passed through the subacute phase of stroke. Despite these promising results, it is crucial to acknowledge the limitations of this study. The small sample size may limit the generalizability of the findings, and future studies should focus on larger cohorts and long-term follow-up to further validate the sustained effects of such therapeutic approaches. Long-term patient monitoring will be essential to assess the durability of the effects of the multicomponent educational-rehabilitative treatment and its impact on quality of life. Furthermore, the lack of detailed data regarding the severity of stroke and disability, such as NIHSS and mRS scores, complicates accurate participant assessment, while the absence of follow-up regarding brain lesion lateralization further extends the study’s limitations.

## Conclusion

Analysis of the results after twenty sessions of educational-rehabilitative treatment in the subacute phase of stroke revealed significant improvements in cognitive functions, motor abilities of the affected upper extremity, and independence in performing daily activities, compared to the control group. These findings underscore the critical importance of applying a holistic approach in the assessment, planning, and implementation of rehabilitation treatment, highlighting the need for an integrated approach in stroke rehabilitation processes.
